# Heterotic effects on litter traits in crossbreeding experiment involving Egyptian rabbit lines

**DOI:** 10.1007/s11250-025-04305-x

**Published:** 2025-03-06

**Authors:** Abdelwahab H. Khattab, Mahmoud M. Iraqi, Maher H. Khalil, Emad M. Amin, Ayman G. EL Nagar

**Affiliations:** 1 Department of Animal Production, Faculty of Agriculture at Moshtohor, Benha University, Tukh, 13736 Egypt; 2 Department of Animal and Poultry Breeding, Animal and Poultry Production Division, Desert Research Center, Ministry of Agriculture, El-Matareya 11753, Cairo, Egypt

**Keywords:** Rabbits, Crossbreeding, Pre-weaning litter traits, Heritability, Direct additive and maternal effects, Direct and maternal heterosis

## Abstract

A four-years crossbreeding experiment was performed involving two synthetic rabbit lines of APRI (A) and Moshtohor (M) where bucks of the APRI line were mated with does of the Moshtohor line to produce F_1_ crossbred (½A½M), followed by inter-se mating to obtain F_2_ crossbred (½A½M)^2^. A total of 669 litters produced from 184 bucks and 394 does were used in analyzing litter size at birth (LSB) and weaning (LSW), litter weight at birth (LWB) and weaning (LWW) and pre-weaning mortality (PM). Heritability estimates for the targeted traits were low to moderate, ranging from 0.11 to 0.27. APRI line had the lowest range in predicted breeding values (PBVs) for all litter traits. The ranges in PBV for the four genetic groups were high and ranging from 0.59 to 1.0 kit for LSB, 2.4 to 3.0 kit for LSW, 26.5 to 33.9 g for LWB, 411.6 to 515.5 g for LWW and 20.9 to 24.8% for PM. The estimates of direct additive effects (G^I^) and maternal effects (G^M^) for litter traits were in favour of the Moshtohor line with percentages ranging from 1.2 to 16.3% for G^I^ and 1.4 to 20.5% for G^M^. The estimates of direct heterosis (H^I^) and maternal heterosis (H^M^) were significantly positive for most litter traits with percentages ranging from 2.4 to 55.8% for H^I^ and 1.8 to 34.6% for H^M^. In practice, it is recommended to use the APRI line as a sire group and Moshtohor line as a dam group in the crossbreeding program to synthesize new synthetic rabbit lines in Egypt.

## Introduction

Crossbreeding in farm animals aimed to increase fitness and fertility, break down accumulated inbreeding that may have arisen through the selection process, and avoid the negative consequences of inbreeding depression (Adenaike et al. 2013; Bunning et al. 2019). The main goal of any crossbreeding program involving local and exotic rabbit breeds is to take the advantages of local breeds which distinguished by good feed utilization, outstanding immunity against most endemic diseases and high adaptation to hot climate conditions, while exotic breeds/lines are characterized by high fertility and prolificacy, and high milk production (Iraqi et al. 2008; Youssef et al. 2009). Litter size at birth or weaning is the most common selection criteria in maternal lines (Baselga and García 2002; Baselga et al. 2017). Despite having a low heritability, litter size is an essential trait in selection programmes of prolific species (pigs and rabbits), especially in maternal lines; enabling to produce the kits at a reduced cost (Cartuche et al. 2014). Furthermore, litter size at weaning indirectly reflects the does' milk production and maternal abilities and has a genetic determinism similar to that of litter size at marketing (Ragab and Baselga 2011; Ragab et al. 2016). The most crucial traits for an effective production are those related to the doe productivity, including the size and weight of the litters in addition to the ability for milk production, some of these traits have been chosen as selection criteria to create maternal lines of rabbits (De Rochambeau et al. 1998; Baselga 2005; Youssef et al. 2008).

In the last decades, many standard breeds or lines of rabbits have been introduced to Egypt (New Zealand White, Californian, Bouscat, V-Line and Hyplus line, etc*.*) to create highly productive synthetic lines of rabbits through crossing between standard and local breeds/lines (Khalil 2010). Accordingly, some genetic attempts were conducted to establish new lines in Egypt through mating Baladi Black bucks with V-line does along with selection for several generations to produce Alexandria line (El-Raffa 2007), mating Baladi Red bucks with V-line does along with three consecutive generations of selection for litter weight at weaning to produce APRI line (Youssef et al. 2009), and mating Sinai Gabali bucks with V-line does to produce Moshtohor line along with four generations of selection for litter weight at weaning and individual weight at 56 days (Iraqi et al. 2010a, b). However, the reviewed estimates of direct additive and/or maternal effects for litter traits at birth and weaning in the Egyptian crossbreeding studies were significantly in favour of NZW, V-line, Hyplus, and Sinai Gabali breeds relative to the other breeds/lines (Khalil et al. 1995, 2002, 2005; Khalil and Afifi 2000; Iraqi et al. 2006; Youssef et al. 2008; Sanad 2023). In addition, desirable and significant direct and maternal heterotic effects on pre-weaning litter traits were verified in several crossbreeding experiments (Khalil et al. 1995, 2002; Khalil and Afifi 2000; Baselga et al. 2003; Brun and Baselga 2005; Iraqi et al. 2006, 2007, 2010b; Attalah et al. 2007; Youssef et al. 2008; Ragab et al. 2016; Sanad 2023). However, the studies concerning the evaluation of direct additive and maternal effects and direct and maternal heterotic effects for litter traits in crossbreeding experiments involving new synthetic rabbit lines in Egypt such as APRI, Moshtohor and Alexandria rabbit lines (Abou Khadiga et al. 2012) are scarce. Therefore, the main objective of the present study was to estimate some crossbreeding effects of direct additive effects, maternal effects, and direct and maternal heterotic effects on pre-weaning litter traits in a crossbreeding experiment of mating bucks of APRI rabbit line with does of Moshtohor rabbit line and their F_1_ and F_2_ crosses.

## Materials and methods

### Ethical statement

All experimental procedures involving animal handling and treatment were approved by the Research Ethics Committee of the Faculty of Agriculture, Benha University, Egypt (Approval No. REC-FOABU 0.3/00029).

### Animals and management

A four-years crossbreeding experiment was performed in hot climate country between bucks of APRI rabbit line (A) and does of Moshtohor line (M) to produce F_1_ crossbred rabbits (½A½M), followed by *inter-se* mating to obtain F_2_ crossbred (½A½M)^2^, consequently, four genetic groups were used in this study. The sub-tropical environment in which the temperatures is higher than 35 °C are accompanied by high relative humidity which is normally over 85% during the day and can reach 100% during the night hot months in Egypt; Marai et al. 2006). The total numbers of bucks, does and litter used in this experiment were presented in Table 1. The experimental work was started from September 2016 to June 2020 in the rabbitry belonging to the Faculty of Agriculture, Benha University, Egypt. The breeding bucks and does were first mated at the age of 18 to 20 weeks. Using natural mating, bucks were divided into different families, each family consisted of one buck and three does; avoiding the matings between animals with common parents. Throughout the duration of the experiment, all the animals were housed in the farm under the same managerial system, feeding regime and hygiene conditions described by Khattab et al. (2024). The litters were weaned after four weeks from kindling.

**Table 1:** Genetic groups and numbers of bucks, does, and litters born and weaned (between brackets) used in the experiment

Buck genetic group	Doe genetic group	Litter genetic group	No. of litters born	No. of litters weaned
A (57)	A (114)	A	164	124
M (52)	M (117)	M	158	128
A (38)	M (75)	½A½M	163	160
½A½M (37)	½A½M (88)	(½A½M)^2^	184	178
Total No. 184	394		669	590

*A* APRI line, *M* Moshtohor line

### Data and model of analysis

Data of pre-weaning litter traits in terms of litter size at birth (LSB) and weaning (LSW), litter weight at birth (LWB) and weaning (LWW) and pre-weaning mortality (PM) were recorded for each litter of the doe in the four genetic groups studied. Prior to the data analysis, the pedigree file was examined for relationship issues (*i.e.* errors in identification number, duplicated animal ID’s, male and female with the same ID, the inbreeding coefficients of the pedigreed animals) using the CFC software version 1.0 (Sargolzaei et al. 2006). Then, the animals were renumbered and the data in the pedigree file was recoded using the renumf90 software (Misztal et al. 2018). The variance components for the random effects and heritabilities of the studied traits were estimated by repeatability single-trait animal model based on a Bayesian inference of Gibbs Sampling Algorithm using the TM software where the Gibbs sampler algorithm comprised 200,000 iterations and discarding the first 20,000 iteration. Afterwards, one sample in each 200 iterations was saved (Legarra et al. 2008):

$${\varvec{y}}={\varvec{X}}{\varvec{b}}+{{\varvec{Z}}}_{{\varvec{a}}\boldsymbol{ }}{{\varvec{u}}}_{{\varvec{a}}\boldsymbol{ }+}{{\varvec{Z}}}_{{\varvec{p}}}{{\varvec{u}}}_{{\varvec{p}}}+{\varvec{e}}\boldsymbol{ }\boldsymbol{ }\boldsymbol{ }{\varvec{M}}{\varvec{o}}{\varvec{d}}{\varvec{e}}{\varvec{l}}1$$

where $y$ = vector of the observed litter trait for the doe rabbit; $b$ = vector of the fixed effects of the genetic group of the doe (4 levels; A, M, ½A½M and (½A½M)^2^), year-season at kindling (16 levels: 4 seasons each year), and parity order (6 levels); ${u}_{a}$= vector of random additive genetic effects of the doe; ${u}_{p}$= vector of non-additive random permanent environmental effects; and $X, {Z}_{a } \text{and}$ ${Z}_{p}$ are design matrices corresponding to the fixed effects, random additive genetic effects and permanent environmental effects, respectively; $e$ = vector of random residual effects of the model. The variance components estimated by Bayesian Gibbs Sampling were consequently used to solve the mixed model equations using the PEST software (Groeneveld 2006), getting the solutions for the fixed effects of genetic groups and their error variance–covariance matrix. Subsequently, the BLUPF90 software (Misztal et al. 2018) was used to estimate the predicted breeding values (PBVs) for each animal adopting the same repeatability single-trait animal model (Model 1). The correlations between the true and predicted breeding values were used to define the accuracy of PBVs (${r}_{A}$). The accuracy of PBV was calculated for each animal as: ${r}_{A}=\sqrt{1-(PEV/{\sigma }_{a}^{2})}$, where PEV is the prediction error variance estimated as PEV = (SEP)^2^ and SEP is the standard error of prediction and ${\sigma }_{a}^{2}$ is the additive genetic variance of the trait.

### Estimation of crossbreeding effects

The crossbreeding effects in terms of direct additive genetic effects, maternal effects, direct heterosis and maternal heterosis for pre-weaning litter traits were estimated using the CBE software (Wolf 1996). The solutions of the crossbreeding effects for the genetic group were obtained according to the Dickerson model (Dickerson 1992), using the procedure of Generalized Least Squares and applying the following linear model:

$$\mathbf{y}=\mathbf{X}\mathbf{b}+\mathbf{e},\mathbf{V}\mathbf{a}\mathbf{r}\left(\mathbf{y}\right)=\mathbf{V}{\varvec{M}}{\varvec{o}}{\varvec{d}}{\varvec{e}}{\varvec{l}}\boldsymbol{ }2$$

where: $\mathbf{y}$= vector of the genetic group solutions of litter trait; $\mathbf{X}$= incidence matrix of genetic group effects; $\mathbf{b}$ = vector of estimable crossbreeding genetic effects; $\mathbf{e}$ = vector of random error; $\mathbf{V}$= the error variance–covariance matrix of $\mathbf{y}$.

The differences between the genetic groups were estimated considering the crossbreeding parameters in terms of direct additive effects (G^I^ = G^I^_A_ − G^I^_M_), maternal effects (G^M^ = G^M^_A_ − G^M^_M_), direct heterosis (H^I^) and maternal heterosis (H^M^). Thus, we have four parameters and these parameters were estimated according to Dickerson (1992) and Wolf (1996) as a vector called b-vector:

$${\varvec{b}}=\left[\left({{{\varvec{G}}}^{{\varvec{I}}}}_{{\varvec{A}}}-{{{\varvec{G}}}^{{\varvec{I}}}}_{{\varvec{M}}}\right)\left({{{\varvec{G}}}^{{\varvec{M}}}}_{{\varvec{A}}}-{{{\varvec{G}}}^{{\varvec{M}}}}_{{\varvec{M}}}\right){{\varvec{H}}}^{{\varvec{I}}}{{\varvec{H}}}^{{\varvec{M}}}\right]$$

where G^I^_A_ and G^I^_M_ = Direct additive genetic effects for APRI and Moshtohor lines, respectively; G^M^_A_ and G^M^_M_ = Maternal effects for APRI and Moshtohor lines, respectively. The solutions of ${\varvec{b}}$ were calculated by the method of Generalized Least Squares using the following equation: $\widehat{{\varvec{b}}}=({{\varvec{X}}}^{\mathbf{^{\prime}}}{{\varvec{V}}}^{-1}{\varvec{X}}{)}^{-}{{\varvec{X}}}^{\mathbf{^{\prime}}}{{\varvec{V}}}^{-1}{\varvec{y}}$ where $\mathbf{X}$ was the matrix of the coefficients relating the means of the genetic groups with the estimable crossbreeding effects (Table 2), V^−^ = the generalized error variance–covariance matrix, with the variance–covariance matrix of the estimate of b, being: ${\varvec{V}}{\varvec{a}}{\varvec{r}}\boldsymbol{ }\widehat{{\varvec{b}}}=({{\varvec{X}}}^{\mathbf{^{\prime}}}{{\varvec{V}}}^{-1}{\varvec{X}}{)}^{-}$.

**Table 2:** Genetic groups of bucks, does and litters and coefficients of the matrix relating the generalized least square means of the genetic groups with the estimable crossbreeding effects

Genetic groups			Mean	Coefficients of the matrix of the estimable crossbreeding effects					
Buck	Doe	Litter		G^I^_A_	G^I^_M_	G^M^_A_	G^M^_M_	H^I^	H^M^
A	A	A	1	1	0	1	0	0	0
M	M	M	1	0	1	0	1	0	0
A	M	½A½M	1	0.5	0.5	0	1	1	0
½A½M	½A½M	(½A½M)^2^	1	0.5	0.5	0.5	0.5	0.5	1

*A* APRI rabbit line, *M* Moshtohor rabbit line, *G*^I^_A_ and *G*^I^_M_ Direct additive genetic effects for APRI and Moshtohor lines, respectively, *G*^M^_A_ and G^M^_M_ Maternal effects for the APRI and Moshtohor lines, respectively, H^I^ Direct heterosis, H^M^ Maternal heterosis

## Results and discussion

### Descriptive statistics for litter traits

The generalized least square means (GLM), standard deviations (SD) and coefficients of variation (CV%) for pre-weaning litter traits across all the genetic groups are shown in Table 3. The GLM for LSB, LSW, LWB, LWW and PM were 7.5 kit, 4.9 kit, 387 g, 2533 g, and 34.7%, respectively. These means were in accordance with the previously concerned studies in Egypt involving the V-line, APRI line and Moshtohor line (Iraqi et al. 2007, 2010b; Youssef et al. 2008; Abou Khadiga et al. 2012; Abdel-Kafy et al. 2012; Rabie et al. 2019).

**Table 3:** Descriptive statistics in terms of generalized least square means (GLM), standard deviations (SD), minimum and maximum values, and coefficient of variation (CV%) for pre-weaning litter traits across all genetic groups

**Trait**^+^	**N**	**GLM**	**SD**	**Min**	**Max**	**CV%**
LSB, kit	669	7.5	1.9	2	13	25
LSW, kit	590	4.9	2.8	1	11	58
LWB, g	669	387	92.4	135	675	24
LWW, g	590	2533	1494	380	5805	59
PM, %	587	34.7	20.20	8	78	58

^+^*LSB* litter size at birth, *LSW* litter size at weaning at 28 days, *LWB* litter weight at birth, *LWW* litter weight at weaning at 28 days, *PM* pre-weaning mortality

The coefficients of variation (CV%) for litter traits were moderate to high and ranged from 24 to 59%, indicating that these estimates increased from birth to weaning (Table 3). These estimates for litter traits agreed with those reported by some Egyptian studies, ranging from 12.4 to 59.8% in New Zealand rabbits (Iraqi 2008), 21.1 to 41.4% in Californian and Baladi Red rabbits (Gharib et al. 2008) and 33.3 to 41.2% in Baladi Black rabbits (Abdel-Kafy et al. 2012).

### Heritability estimates and permanent environmental effects

Heritability estimates for litter traits were low or moderate and ranged from 0.11 to 0.27 (Table 4). These estimates were in accordance with those estimates previously reported in Egyptian studies (Iraqi et al. 2006; Rabie et al. 2019; Behiry et al. 2021; Gharib et al. 2022; Sanad 2023). During the period from birth to weaning, the litters were sensitive to environmental and management changes, and consequently, most genetic improvements in litter traits can be achieved through the environmental enhancement of postnatal litter management. Iraqi et al. (2006) reported that small estimates of heritability for litter traits might be due to the large maternal effects and/or variation due to permanent environmental effects, i.e. increasing non-additive genetic effects. Additionally, the low heritability estimates for litter traits may be attributed to the negative correlation between milking, maternal abilities, and direct effects of the doe (Iraqi et al. 2010b). Furthermore, it was observed that heritability estimates for litter size and litter weight traits increased with the advance of age from birth to weaning (Table 4) as previously reported by Khalil et al. (1987) who stated that selection for litter size and weight at weaning could be of considerable importance rather than at birth.

**Table 4:** Estimates of heritability (*h*^*2*^ ± SE), permanent environmental effects (*p*^*2*^ ± SE) and random error (*e*^*2*^ ± SE) for pre-weaning litter traits across all genetic groups

Trait^+^	N	*h*^*2*^ ± SE	*p*^*2*^ ± SE	*e*^*2*^ ± SE
LSB	669	0.18 ± 0.09	0.09 ± 0.08	0.73 ± 0.09
LSW	590	0.27 ± 0.11	0.07 ± 0.07	0.66 ± 0.09
LWB	669	0.11 ± 0.07	0.07 ± 0.06	0.82 ± 0.08
LWW	590	0.24 ± 0.11	0.08 ± 0.07	0.68 ± 0.10
PM	587	0.15 ± 0.10	0.18 ± 0.11	0.76 ± 0.10

^+^*LSB* litter size at birth, *LSW* litter size at weaning at 28 days, *LWB* litter weight at birth, *LWW* litter weight at weaning at 28 days, *PM* pre-weaning mortality

The proportion of phenotypic variance explained by the permanent environmental effects (*P*^*2*^) for pre-weaning litter traits were small and ranged from 0.07 to 0.18 (Table 4), indicating that the permanent environmental effects were small and negligible in their mothering ability. Similarly, Nagy et al. (2014) reported that the permanent environmental effects had smaller impacts on pre-weaning litter traits compared with the additive genetic effects in the Pannon Ka synthetic rabbit line. Furthermore, Piles et al. (2006) stated that the *P*^*2*^ ratios ranged from 0.03 to 0.10 for the total number of kits born (TNB), number born alive (NBA) and number of kits weaned (NW) in A-line, Prat line, and V-line rabbits. Ragab and Baselga (2011) in the four maternal specialized rabbit lines (A, V, H, and LP), reported that the *P*^*2*^ ratios ranged from 0.08 to 0.10 for the three aforementioned traits (TNB, NBA and NW).

## Predicted breeding values (PBV)

In all genetic groups, the range in PBV and their accuracies for pre-weaning litter are shown in Table 5. APRI line had the lowest PBV for all traits studied. In general, the ranges in PBV for litter traits among the four genetic groups were high and ranged from 0.6 to 1.0 kit for LSB, 2.4 to 3.0 kit for LSW, 26.5 to 33.9 g for LWB, 411.6 to 515.5 g for LWW and 20.9 to 24.8% for PM (Table 5). The reviewed ranges of the PBV from the Egyptian studies were high and ranged from 0.22 to 3.23 kit for litter size at birth, 1.02 to 3.21 kit for litter size at weaning, 23 to 400 g for litter weight at birth, and 164 to 880 for litter weight at weaning (Rabie et al. 2019; Behiry et al. 2021; Hassan et al. 2022). Shehab El-Din (2022) found that the PBV for LSB, LSW, LWB and LWW 2.36 kit, 1.41 kit, 91 g and 638 g, respectively in V-line rabbits.

**Table 5:** The ranges in predicted breeding values (PBV) for pre-weaning litter traits in each separate genetic group

Trait^+^	Genetic group							
	APRI line (A)		Moshtohor line (M)		½A½M		(½A½M)^2^	
	N	PBV range	N	PBV range	N	PBV range	N	PBV range
LSB, kit	164	0.6	158	0.75	163	0.76	184	1.0
LSW, kit	124	2.4	128	2.5	160	3.0	178	2.8
LWB, g	164	26.5	158	28.5	163	30.9	184	33.9
LWW, g	124	411.6	128	515.5	160	459.3	178	420.1
PM, %	161	20.9	152	24.8	132	21.7	142	21.2

^+^*LSB* litter size at birth, *LSW* litter size at weaning at 28 days, *LWB* litter weight at birth, *LWW* litter weight at weaning at 28 days, *PM* pre-weaning mortality, the accuracies of predictions in PBV were high and ranged from 0.61 to 0.85 for pre-weaning litter traits

### Direct additive and maternal effects

The generalized least square solutions (GLS) of the direct additive genetic effects (G^I^) and maternal effects (G^M^) measured in units and percentages for pre-weaning litter traits were in favour of the Moshtohor line with percentages ranging from 1.2 to 16.3% for G^I^ effects and 1.4 to 20.5% for G^M^ effects (Table 6). However, the reviewed estimates cited in the Egyptian studies indicated that direct additive genetic effects and maternal effects on litter traits at birth and weaning were significantly in favour of NZW, V-line, Hyplus, and Sinai Gabali breeds relative to the other breeds/lines (Khalil et al. 1995, 2002, 2005; Khalil and Afifi 2000; Iraqi et al. 2006; Youssef et al. 2008; Sanad 2023). These reviewed percentages were mostly moderate and ranged from 10.0 to 39.8% for LSB, 7.0 to 24.9% for LSW, 4.0 to 42.9% for LWB, 3.0 to 15.5% for LWW, and 1.4 to 31.8% for PM. Al-Saef et al. (2008) in crossing Saudi Gabali with V-line rabbits found that G^I^ effects were significant for all litter traits (12.3 to 31.8%) and neither maternal nor grand-maternal effects had a significant impact on litter and milk yield traits. García et al. (2012) in a complete diallel crossbreeding between four rabbit genetic groups in Cuba (California, Chinchilla, New Zealand White, and Semigiant), revealed that there were significant differences in G^I^ between the genetic groups, favoring NZW rabbits and demonstrated that maternal effects were more important than direct genetic effects for litter traits studied. In contrast to the present results, Baselga et al. (2003) in crossing three Spanish maternal rabbit lines (A, V, and H), reported that the differences between the genetic groups due to maternal effects for LSB and NBA were important, and favoring V-line does. In crossing Saudi Gabali and V-line rabbits, Khalil et al. (2005) reported that percentages of G^M^ were significantly in favour of V-line does, ranging from 3.1 to 15.7% for litter size and weight at birth and weaning. In contrast, in a diallel crossbreeding involving four Spanish specialized maternal lines of rabbits, Ragab et al. (2016) reported that these maternal lines generally did not reveal any changes in direct genetic effects for litter size at birth and weaning.

**Table 6:** Generalized least square solutions (GLS) and their percentages of direct additive effects (G^I^) and maternal effects (G^M^) for pre-weaning litter traits in crossing APRI bucks with Moshtohor does

Trait^+^	Direct additive genetic effects (G^I^ = G^I^_A_-G^I^_M_)				Maternal effects (G^M^ = G^M^_A_-G^M^_M_)		
	N	In units	SE	As %^++^	In units	SE	As %^++^
LSB, kit	669	−0.2*	0.01	2.8	−0.5**	0.01	7.0
LSW, kit	590	−0.4**	0.01	10.2	−0.8**	0.01	20.5
LWB, g	669	−4.5^NS^	0.20	1.2	−5.3^NS^	0.20	1.4
LWW, g	590	−151*	3.00	7.6	−396**	2.50	19.8
PM, %	587	9.5**	0.10	16.3	8.7**	0.10	14.9

^+^*LSB* litter size at birth, *LSW* litter size at weaning at 28 days, *LWB* litter weight at birth, *LWW* litter weight at weaning at 28 days, *PM* pre-weaning mortality, *SE* standard errors, ^**++**^Percentage computed as [Estimate of G^I^ or G^M^ in units/(A + M)/2] × 100; *NS* Not Significant, * = *P* ≤ 0.05 and ** = *P* ≤ 0.01

### Direct and maternal heterosis

The GLS of the direct heterosis (H^I^) and maternal heterosis (H^M^) as well as their percentages for pre-weaning litter traits presented in Table 7 indicated that the estimable solutions of H^I^ and H^M^ were significant for most traits studied whereas the percentages ranged from 2.4 to 55.8% in case of direct heterosis and 1.8 to 34.6% in case of maternal heterosis. However, significant H^I^ on pre-weaning litter traits indicates that crossbreed does and/or crossbred dams of does were associated with favorable heterotic effects on litter traits in terms of larger litter size at birth and weaning, heavier litter weight at birth and weaning along with lesser pre-weaning mortality. However, direct heterosis as evidenced in most of the crossbred does and/or crossbred dams obtained in several Egyptian studies (e.g. Khalil and Afifi 2000; Khalil et al. 2002; Attalah et al. 2007; Iraqi et al. 2006, 2007, 2010; Youssef et al. 2008; Sanad 2023). These reviewed percentages were mostly moderate and ranged from 10.1 to 23.9% for LSB, 12.0 to 27.7% for LSW, 9.0 to 27.6% for LWB, 3.0 to 27.6% for LWW and −4.1 to −27.5% for PM. For maternal heterosis, the percentages cited in the Egyptian studies were mostly positively moderate and ranged from 3.0 to 10.0% for LSB, 8.1 to 11.4% for LSW, 6.1 to 10.0% for LWB, 2.9 to 12.0% for LWW and 2.0 to 12.3% for PM (Khalil et al. 2002; Youssef et al. 2008; Iraqi et al. 2006, 2010a). In a crossbreeding experiment between Saudi Gabali and V-line rabbits, Khalil et al. (2005) reported that estimates of maternal heterosis were significantly favorable for LSB, LSW, and LWW, being 0.81 kit, 0.74 kit, and 170 g, respectively. Moreover, Al-Saef et al. (2008) in crossing Saudi Gabali and V-line rabbits, found that maternal heterosis were 0.73 and 0.72 kit for LSB and LSW, respectively.

**Table 7:** Generalized least square solutions (GLS) and their percentages of direct heterotic effects (H^I^), and maternal heterotic effects (H^M^) for pre-weaning litter traits in crossing APRI bucks with Moshtohor does

Trait^+^	N	Direct heterotic effects (H^I^)			Maternal heterotic effects (H^M^)		
		In units	SE	As %^++^	In units	SE	As %^++^
LSB, kit	669	1.3**	0.01	18.3	0.13^NS^	0.01	1.8
LSW, kit	590	1.9**	0.01	48.7	0.8**	0.01	20.5
LWB, g	669	9.0^NS^	0.30	2.4	10.7^NS^	0.30	3.0
LWW, g	590	1113**	4.90	55.8	431**	4.60	21.6
PM, %	587	−9.8**	0.10	16.8	−20.2**	0.10	34.6

^+^*LSB* litter size at birth, *LSW* litter size at weaning at 28 days, *LWB* litter weight at birth, *LWW* litter weight at weaning at 28 days, *PM* pre-weaning mortality, *SE* standard errors, ^**++**^Percentage computed as [Estimate of H^I^ or H^M^ in units/(A + M)/2] × 100; *NS* Not Significant and ** = *P* ≤ 0.01

## Conclusion

Desired direct and maternal heterotic effects have been evidenced in this crossing experiment between APRI and Moshtohor rabbit lines. Therefore, crossing APRI and Moshtohor rabbit lines was associated with beneficial consequences in pre-weaning litter and prolificacy traits. In this concept, bucks from the APRI rabbit line could be used as sires, whereas does from the Moshtohor rabbit line could be used as dams to develop new rabbit lines with acceptable prolificacy and productive performance in Egypt. Our findings may serve as encouraging factors for the rabbit breeders in Egypt and other hot-climate countries to involve APRI and Moshtohor rabbit lines in their crossbreeding programs.

## Data Availability

Available upon reasonable request from the first author.
